# The Future of Research in Tourette Syndrome

**DOI:** 10.3233/BEN-120297

**Published:** 2013

**Authors:** Andrea E. Cavanna, Conor Kavanagh, Mary M. Robertson

**Affiliations:** ^1^Department of NeuropsychiatryUniversity of Birmingham and BSMHFTBirminghamUK; ^2^Sobell Department of Motor Neuroscience and Movement DisordersInstitute of NeurologyUCLLondonUK; ^3^Department of Mental Health SciencesUCLLondonUK; ^4^Department of NeurologySt George’s Hospital and Medical SchoolLondonUK

**Keywords:** Tourette syndrome, tics, research, phenomenology, pathophysiology, genetics, treatment

## Abstract

Tourette syndrome (TS) is a neurological condition first described by Georges Gilles de la Tourette in 1885. TS was largely thought of as a rare and bizarre condition until the 1960s, when the beneficial effects of neuroleptics on tic symptoms led to an exponential increase in neuroscientific research. Today TS is known to be a relatively common condition that is frequently misdiagnosed due to a combination of its variable manifestation and the waxing and waning of tic frequency and severity. Although there has been a paucity of research on TS compared to other movement disorders, in recent years TS has garnered increasing interest and has shown a number of novel and complex sides, about which much is yet to be learnt. The present article discusses where research has taken us thus far and where it is heading in all the major facets of this fascinating condition.

## 1. Introduction

Tourette syndrome (TS) has fascinated clinicians and researchers alike over the decades [1,2], but has attracted relatively little research until recently [3]. This article focuses on where this research has taken us and where it is heading.

The first important area of research in TS is about the clinical phenomenology and the relationship between tics and behavioural symptoms. TS is commonly associated with attention deficit and hyperactivity disorder (ADHD), obsessive-compulsive disorder (OCD), depression and personality disorders and in fact clinical studies conducted both in specialist clinics [4,5] and in the wider community [6] have consistently shown that only 8–12% of patients with TS have no other psychopathology [7]. Research has been performed to determine the nature of the relationship between TS and these co-morbidities and has suggested that specific obsessive-compulsive symptoms can represent an alternative phenotypic expression of TS, whilst depression seems to be multifactorial in origin, with a major contribution by the co-morbidity with OCD and ADHD rather than TS itself. Of course there remain unclear points, for example whether tic disorders plus ADHD represent a separate nosological entity, a combination of two independent pathologies or a phenotype subgroup of either ADHD or TS. Also, the exact nature of the relationship between personality disorders and TS remains unknown [8].

The term endophenotype was coined in 1967 by Gottesman and Shields [9] to refer to a biological trait that is an intermediary between certain genes and a pattern of behavior. Exciting new studies are currently being conducted to find endophenotypes in the field of TS and tic disorders. For example, it has been suggested that pre-frontal cortex thinning and reduction in caudate volume may be endophenotypes for TS, as they correlate with tic severity [10]. Another example is the fine-motor skills demonstrated on the Purdue Pegboard test, which may also serve as a helpful endophenotype by giving a rough estimate of the level of basal ganglia dysfunction [11].

One of the major challenges for clinicians who see children with TS is to be able to predict the clinical

ISSN 0953-4180/13/$27.50 © 2013 – IOS Press and the authors. All rights reserved course or long-term outcome of the condition. Tics generally appear between the ages of 4–6, reach their worst-ever severity around the ages of 10 and 12 and in the vast majority of patients improve during the course of adolescence [12]. Although it appears that the presence of co-morbid conditions in childhood correlate poorly with tic severity in adulthood [13,14] putative endophenotypes such as poor fine motor skills [11] and smaller caudate volumes in childhood seem to be associated with increased adulthood tic severity [15]. Neuroimaging data also suggest that certain regions in the brain may be involved in persistent TS, however further longitudinal studies are required to confirm this [16]. Despite these preliminary findings, there are still no clinical measures that can allow us to reliably predict whose symptoms will persist into adulthood.

The pathophysiology of TS is still largely unknown. Research suggests that the dorsolateral pre-frontal cortex (DLPFC) [17] and the basal ganglia (in particular the caudate region [18] which receives input from the DLPFC [19]) are involved in the pathogenesis of TS. More recently, post-mortem studies have implicated a deficiency of parvalbumin striatal interneurons in the development of TS symptoms [20]. Gene-environment interaction studies on the development of parvalbumin interneurons are ongoing [21] and will likely provide a better understanding of the causes of TS and assist the development of new treatments in the future.

Various techniques and methodologies are currently being used to investigate the pathophysiology of TS. For instance, structural and functional imaging have found evidence for compensatory neuroplastic changes (particularly in the prefrontal cortex and corpus callosum), possibly reflecting active efforts to gain control of the tic symptoms [22,23]. Neurophysiological investigations have demonstrated negative potentials occurring 100ms before tic expression, suggesting that simple motor tics might originate from deep brain structures such as the striatal centres [24]. Animal models are receiving increasing attention to examine the pathophysiology of TS. In one study, sera from patients with TS containing anti-neural and anti-nuclear antibodies were injected into rats resulting in oral stereotypies, a finding consistent with autoimmune etiology in a subset of patients with TS [25].

The genetic aspects of TS have proven unexpectedly challenging. Segregation analyses have suggested multiple inheritance patterns in TS [26], and other genetic studies have identified a number of candidate regions which are currently being investigated [27,28]. Although rare, these mutations can make a key contribution to our understanding of disease pathogenesis in TS. TS was once thought to be a unitary condition [29], but recent clinical studies using statistical techniques such as principal-component factor analysis, hierarchical cluster analysis and latent class analysis have suggested there may be multiple distinct TS phenotypes [30]. In order to increase the likelihood of finding genes responsible for TS precise phenotypic definition should be incorporated in future genetic studies.

Despite the number of available therapeutic options, the treatment of TS is still suboptimal. The majority of treatment options for tics are pharmacological [31, 32] although non-pharmacological treatments have increasingly been advocated. These include behavioural techniques, such as Habit Reversal Therapy (HRT) and Exposure and Response Prevention (ERP) [33], and stereotactic neurosurgery, namely Deep Brain Stimulation (DBS). Randomized controlled studies comparing the efficacy of behavioral techniques to pharmacological therapies are needed. Moreover, although HRT has shown a durable response over time, long-term studies are currently lacking for ERP [34]. Treatment-resistant patients whose tics significantly impair their health-related quality of life may be candidates for DBS (usually targeting the thalamus or globus pallidus – pars interna), which has shown to have the potential to improve both tics and psychiatric co-morbidities [35]. Larger randomized controlled trials are needed to confirm the efficacy of specific DBS targets along with their safety, as, although rare, adverse effects such as sedation and changes in sexual function have been reported [36].

Finally, a few recent studies have made insightful contributions to our understanding of cognitive, emotional and social aspects of TS, inspired by novel approaches from the field of social cognition [37–41]. Subtle difficulties in theory of mind and other socially relevant skills have been identified and linked to underlying functional abnormalities within the fronto-basal pathways that appear to be involved In tic generation. From a clinical perspective, these findings could explain at least some of the difficulties encountered in everyday life by patients with TS and could complement the rapidly expanding research area on the determinants of quality of life in this complex condition [42–44].

Across both sides of the Atlantic, research has gathered speed over recent years and consequently more is known about TS than ever before. As corollary of the swell in research, more and more questions are being asked for future researchers to tackle in a field that is becoming increasingly fascinating.

## Acknowledgements

The authors are grateful to Tourette Syndrome Association USA and Tourettes Action UK for their ongoing support.
